# The Extract of *Salvia miltiorrhiza* ‘Hongdan’ Attenuates Inflammation in LPS-Activated BV2 Microglia via ERK1/2, JNK, and p38 MAPK Signaling Inhibition

**DOI:** 10.3390/ph19060818

**Published:** 2026-05-23

**Authors:** Suk Ju, Joonyoung Shin, Hyorin Lee, Gwang Joo Jeon, Dongwoon Han, Sungchul Kim

**Affiliations:** 1 Institute for Global Rare Disease Network, Professional Graduate School of Korean Medicine, Wonkwang University, Iksan 54538, Republic of Korea; christineju@hanmail.net (S.J.); spm1219@naver.com (J.S.); dlgyfls319@naver.com (H.L.); dwhan@hanyang.ac.kr (D.H.); 2 Jeonbuk Advanced Bio-Convergence Academy (JABA), Wonkwang University, Iksan 54538, Republic of Korea; 3 Department of Biotechnology, Hankyong National University, Anseong 17579, Republic of Korea; jeon5894@gmail.com; 4 Department of Global Health and Development, Hanyang University, Seoul 04763, Republic of Korea

**Keywords:** *Salvia miltiorrhiza*, Hongdan, microglia, nitrite, pro-inflammatory cytokines, MAPK signaling pathways

## Abstract

**Background/Objectives:*** Salvia miltiorrhiza* is a medicinal plant rich in phenolic acids and tanshinones, compounds that have been linked to anti-inflammatory and neuroprotective activities. ‘Hongdan’ is a Korean cultivar characterized by relatively high levels of salvianolic acid B and tanshinone IIA, but its anti-inflammatory activity in microglial cells has not yet been examined. **Methods:** Nitrite production and the mRNA expression of inflammatory mediators (iNOS and COX-2) and pro-inflammatory cytokines (IL-1β, IL-6, and TNF-α) were examined. In addition, activation of MAPK (ERK1/2, JNK, and p38) signaling pathway and expression of the NF-κB regulatory protein IκB-α were analyzed. **Results:** The Hongdan extract inhibited nitrite production and reduced the expression of iNOS and COX-2 in LPS-stimulated BV2 microglial cells. In addition, the expression of IL-1β and IL-6 was markedly reduced, whereas TNF-α was significantly suppressed only at the highest concentration tested. Furthermore, phosphorylation of ERK1/2, JNK, and p38 was significantly inhibited, while IκB-α degradation was not altered. **Conclusions:** These findings demonstrate that the Hongdan extract effectively suppresses LPS-induced inflammatory responses through inhibition of MAPK signaling pathways and may serve as a promising natural therapeutic candidate for neuroinflammatory disorders.

## 1. Introduction

With the rapid transition toward an aging society, the prevalence of neurodegenerative diseases such as Alzheimer’s disease, stroke, and Parkinson’s disease has been steadily increasing worldwide [1,2,3]. These disorders not only severely impair patients’ quality of life but also impose substantial medical, social, and economic burdens. Neuroinflammation has been identified as one of the major pathological mechanisms underlying neurodegenerative diseases, and effective regulation of this inflammatory response is therefore considered a key strategy for disease prevention and treatment [4,5].

Neuroinflammatory responses are primarily mediated by the activation of microglial cells [6,7]. Activated microglia excessively produce pro-inflammatory cytokines, including tumor necrosis factor-α (TNF-α), interleukin-1β (IL-1β), and interleukin-6 (IL-6), as well as inflammatory mediators such as nitrite, inducible nitric oxide synthase (iNOS), and cyclooxygenase-2 (COX-2). This overproduction leads to neuronal damage and cell death [8,9]. Because such inflammatory cascades play a critical role in accelerating the progression of neurodegenerative diseases, there is an urgent need to develop safe and effective therapeutic agents capable of suppressing neuroinflammation. Currently, therapeutic approaches aimed at controlling inflammatory signaling include non-steroidal anti-inflammatory drugs (NSAIDs), corticosteroids, and biological agents targeting inflammatory cytokines (e.g., TNF-α inhibitors). Although these treatments have shown efficacy in alleviating symptoms in certain conditions, their long-term use is limited by adverse effects such as immunosuppression, gastrointestinal toxicity, and drug resistance [10,11]. Consequently, the development of alternative therapeutic strategies with fewer side effects remains a critical unmet need.

To overcome the limitations of conventional therapies, medicinal plants with multi-target anti-inflammatory properties have attracted increasing attention as potential alternative strategies [12,13]. Among them, *Salvia miltiorrhiza* Bunge has been traditionally used for symptoms associated with impaired circulation and for pain relief [14,15,16,17]. *Salvia miltiorrhiza* is a perennial herb belonging to the family Lamiaceae (Labiatae), and its roots are primarily used for medicinal purposes [18,19]. Its major bioactive constituents include phenolic acids and tanshinones, both of which have been reported to exhibit potent anti-inflammatory and antioxidant activities as well as significant neuroprotective effects [20,21,22]. In particular, these bioactive constituents have been reported to exert neuroprotective effects in Alzheimer’s and Parkinson’s disease models by targeting key pathological processes underlying neurodegeneration, including oxidative stress, neuroinflammation, and amyloid-β aggregation [23,24,25].

A Korean cultivar of *Salvia miltiorrhiza*, designated ‘Hongdan’, has recently been introduced through breeding efforts led by the Rural Development Administration (RDA) of Korea [26,27,28]. Earlier studies have characterised this cultivar in terms of agronomic performance and phytochemical profile, indicating that it differs from previously available materials in marker-compound composition and cultivation-related traits. However, its biological activity in neuroinflammatory models has not yet been established.

Accordingly, the present study examined whether a 50% ethanol extract prepared from Hongdan roots suppresses inflammatory responses in lipopolysaccharide-stimulated BV2 microglial cells. We assessed cell viability and nitrite production, quantified the mRNA expression of key inflammatory mediators (iNOS and COX-2) and pro-inflammatory cytokines (TNF-α, IL-1β, and IL-6), and further investigated the involvement of the MAPK and NF-κB signalling pathways to clarify the underlying mechanism of action.

## 2. Results

### 2.1. Quality Control (QC) and Chemical Characterization

**Figure 1:**
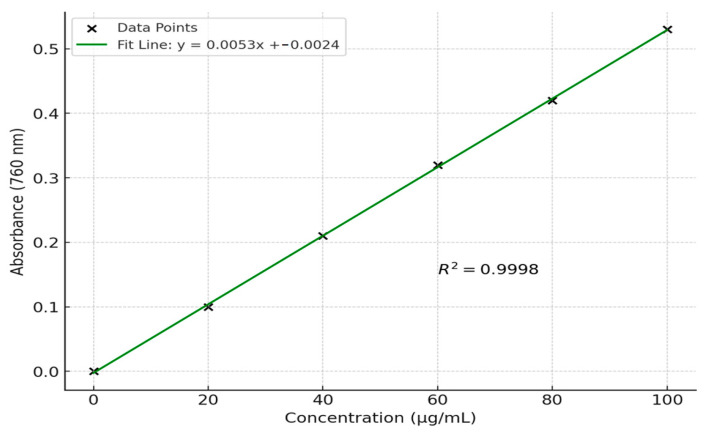
Standard calibration curve of gallic acid used for determination of total phenolic content (TPC) in the Hongdan extract. Absorbance was measured at 760 nm for each concentration, and the results were expressed as μg gallic acid equivalents (GAE)/mL.

**Figure 2:**
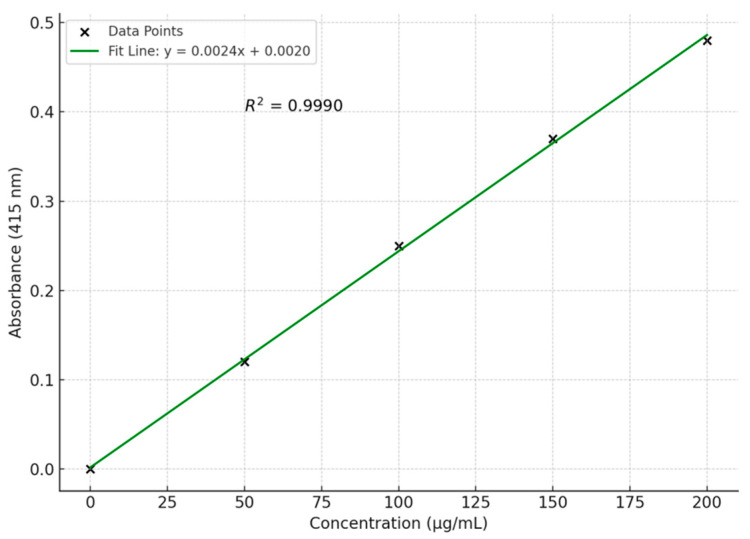
Standard calibration curve of quercetin used for determination of total flavonoid content (TFC) in the Hongdan extract. Absorbance was measured at 415 nm for each concentration, and the results were expressed as μg quercetin equivalents (QE)/mL.

A gallic acid standard calibration curve was generated for determination of total phenolic content (TPC) in the Hongdan extract (Figure 1). Based on this curve, the TPC of the Hongdan extract was 86.24 ± 2.38 μg GAE/mL. Likewise, a quercetin standard calibration curve was generated for determination of total flavonoid content (TFC) (Figure 2), and the TFC was 52.92 ± 5.44 μg QE/mL. For reference, prior HPLC characterization of the Hongdan root material used for extract preparation identified salvianolic acid B and tanshinone IIA as major marker compounds [28]. Salvianolic acid B and tanshinone IIA were reported at 72.35 ± 1.39 mg/g extract and 3.80 ± 0.07 mg/g extract, respectively, with a combined content of 76.15 ± 1.47 mg/g extract (Table 1).

### 2.2. Evaluation of Cytotoxicity of the Hongdan Extract in BV2 Cells

**Figure 3:**
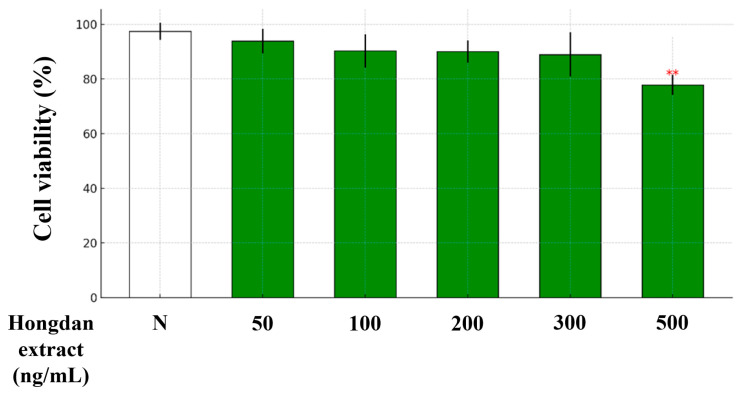
Effect of the Hongdan extract on cell viability in BV2 cells. BV2 cells were incubated with various concentrations of the Hongdan extract (50, 100, 200, 300, and 500 ng/mL). Results are presented as the mean ± standard deviation (SD) of three independent experiments. *, compared with the N group. ** *p* < 0.01.

As shown in Figure 3, the Hongdan extract exhibited no significant cytotoxicity in BV2 cells within the concentration range of up to 300 ng/mL. However, at 500 ng/mL, cell viability was significantly reduced. Therefore, the maximum concentration of the Hongdan extract used in subsequent experiments was set at 300 ng/mL.

### 2.3. Inhibitory Effect of the Hongdan Extract on LPS-Induced Nitrite Production in BV2 Cells

**Figure 4:**
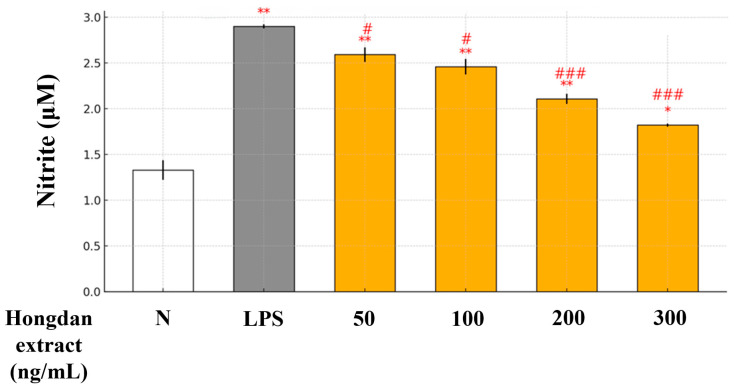
Effects of different concentrations (50, 100, 200, 300 ng/mL) of the Hongdan extract on nitrite production in LPS-stimulated BV2 cells. Results are presented as the mean ± standard deviation (SD) of three independent experiments. *, compared with the N group; #, compared with the LPS group. */# *p* < 0.05, ** *p* < 0.01, ### *p* < 0.001.

To investigate the effect of the Hongdan extract on the inhibition of nitrite production in BV2 cells, cells were pretreated with the extract and then stimulated with LPS. Nitrite levels were markedly elevated in LPS-stimulated BV2 cells compared with the unstimulated control group. Pretreatment with the Hongdan extract significantly and dose-dependently reduced LPS-induced nitrite production at 50, 100, 200, and 300 ng/mL (Figure 4).

### 2.4. Inhibitory Effect of the Hongdan Extract on LPS-Induced iNOS mRNA Expression in BV2 Cells

**Figure 5:**
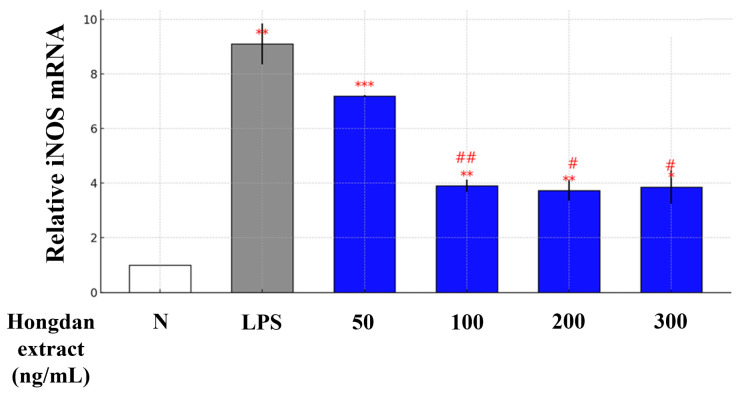
Effects of different concentrations (50, 100, 200, 300 ng/mL) of the Hongdan extract on iNOS mRNA expression in LPS-stimulated BV2 cells. Results are presented as the mean ± standard deviation (SD) of three independent experiments. *, compared with the N group; #, compared with the LPS group. */# *p* < 0.05, **/## *p* < 0.01, *** *p* < 0.001.

To examine whether the Hongdan extract affects the expression of iNOS, an inflammation-mediating inducible enzyme, real-time RT-PCR was performed in BV2 cells stimulated with LPS. BV2 cells were pretreated with the extract for 1 h and subsequently stimulated with LPS for 6 h. Compared with the LPS-stimulated group, treatment with the Hongdan extract significantly reduced the LPS-induced increase in iNOS mRNA expression (Figure 5).

### 2.5. Inhibitory Effect of the Hongdan Extract on LPS-Induced COX-2 mRNA Expression in BV2 Cells

**Figure 6:**
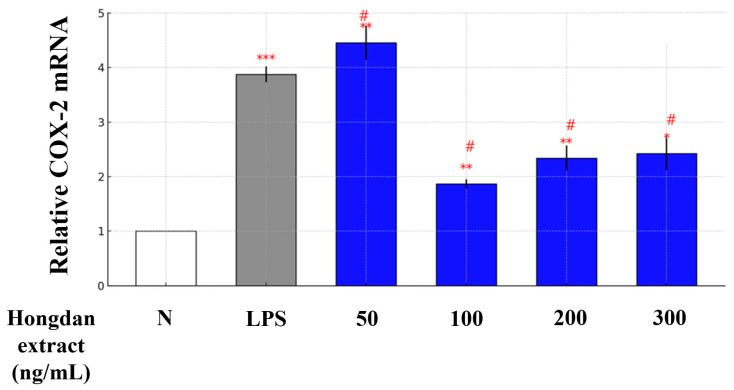
Effects of different concentrations (50, 100, 200, 300 ng/mL) of the Hongdan extract on COX-2 mRNA expression in LPS-stimulated BV2 cells. Results are presented as the mean ± standard deviation (SD) of three independent experiments. *, compared with the N group; #, compared with the LPS group. */# *p* < 0.05, ** *p* < 0.01, *** *p* < 0.001.

To investigate the effect of the Hongdan extract on COX-2 expression during LPS-induced inflammatory responses in BV2 cells, real-time RT-PCR was performed. BV2 cells were pretreated with the extract for 1 h and subsequently stimulated with LPS for 6 h. Treatment with the Hongdan extract significantly reduced the LPS-induced increase in COX-2 mRNA expression (Figure 6).

### 2.6. Inhibitory Effect of the Hongdan Extract on TNF-α Production in BV2 Cells

**Figure 7:**
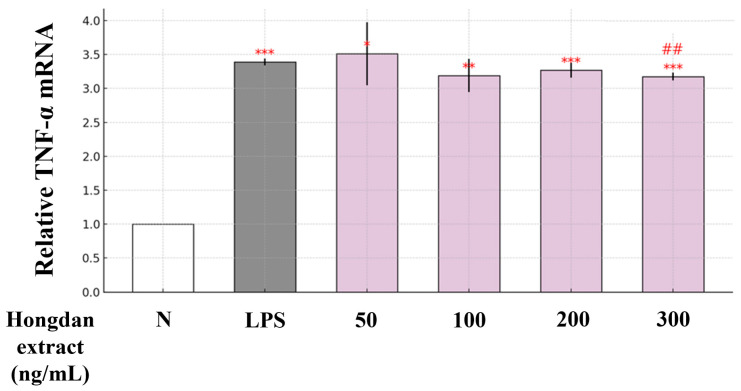
Effects of different concentrations (50, 100, 200, 300 ng/mL) of the Hongdan extract on TNF-α mRNA expression in LPS-stimulated BV2 cells. Results are presented as the mean ± standard deviation (SD) of three independent experiments. *, compared with the N group; #, compared with the LPS group. * *p* < 0.05, **/## *p* < 0.01, *** *p* < 0.001.

To evaluate whether the Hongdan extract could effectively regulate TNF-α, real-time RT-PCR was performed. BV2 cells were pretreated with the extract for 1 h and then exposed to LPS for 6 h to induce inflammation. Although treatment with the Hongdan extract did not markedly suppress the LPS-induced increase in TNF-α mRNA expression at lower concentrations, a statistically significant inhibitory effect was observed at the highest concentration (300 ng/mL) (Figure 7).

### 2.7. Inhibitory Effect of the Hongdan Extract on IL-1β Production in BV2 Cells

**Figure 8:**
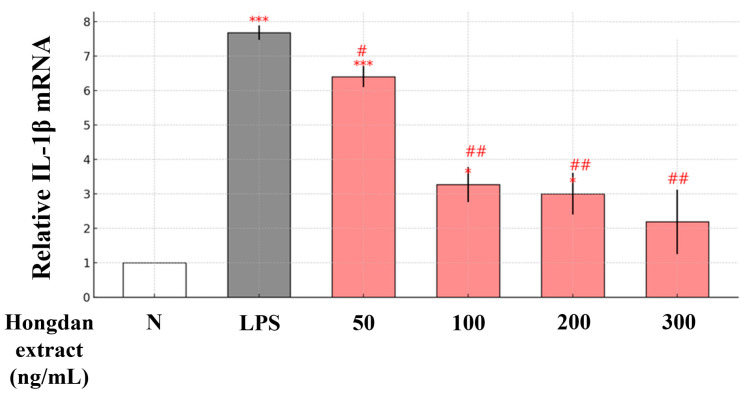
Effects of different concentrations (50, 100, 200, 300 ng/mL) of the Hongdan extract on IL-1β mRNA expression in LPS-stimulated BV2 cells. Results are presented as the mean ± standard deviation (SD) of three independent experiments. *, compared with the N group; #, compared with the LPS group. */# *p* < 0.05, ## *p* < 0.01, *** *p* < 0.001.

To evaluate whether the Hongdan extract could effectively regulate IL-1β, real-time RT-PCR was performed. BV2 cells were pretreated with the extract for 1 h and then stimulated with LPS for 6 h to induce inflammation. Treatment with the Hongdan extract dose-dependently suppressed the LPS-induced increase in IL-1β mRNA expression at all tested concentrations (50, 100, 200, and 300 ng/mL), compared with the control group (Figure 8).

### 2.8. Inhibitory Effect of the Hongdan Extract on IL-6 Production in BV2 Cells

**Figure 9:**
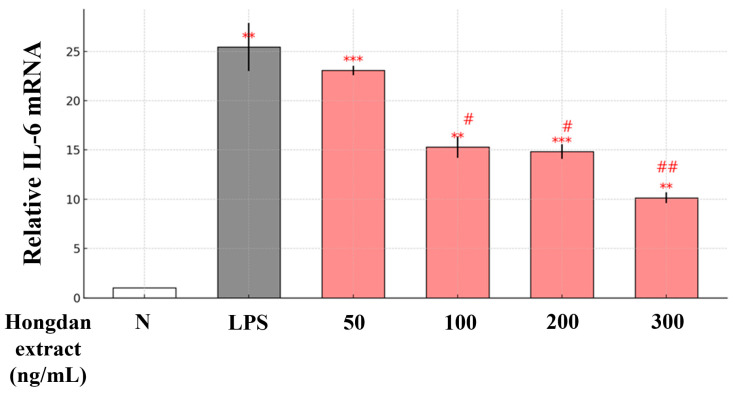
Effects of different concentrations (50, 100, 200, 300 ng/mL) of the Hongdan extract on IL-6 mRNA expression in LPS-stimulated BV2 cells. Results are presented as the mean ± standard deviation (SD) of three independent experiments. *, compared with the N group; #, compared with the LPS group. # *p* < 0.05, **/## *p* < 0.01, *** *p* < 0.001.

To assess whether the Hongdan extract could effectively regulate IL-6, real-time RT-PCR was performed. BV2 cells were pretreated with the extract for 1 h and then stimulated with LPS for 6 h to induce inflammation. Treatment with the Hongdan extract dose-dependently inhibited the LPS-induced increase in IL-6 mRNA expression (Figure 9).

### 2.9. Inhibitory Effects of the Hongdan Extract on MAPK and NF-κB Signaling Pathways in LPS-Induced BV2 Cells

**Figure 10:**
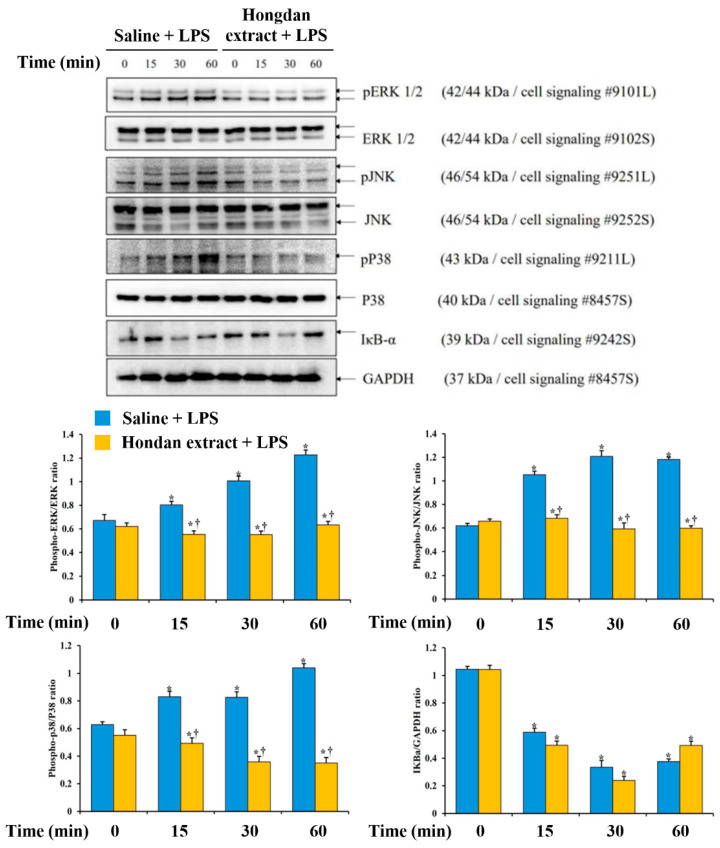
Hongdan extract suppresses MAPK phosphorylation and modulates IκB-α expression in LPS-stimulated BV2 microglia. Representative Western blot images of p-ERK1/2, ERK1/2, p-JNK, JNK, p-p38, p38, IκB-α, and GAPDH in BV2 cells treated with LPS (1 µg/mL) with or without Hongdan extract (300 ng/mL) for the indicated times (0, 15, 30, and 60 min). The graphs below show densitometric quantification of the p-ERK1/2/ERK1/2, p-JNK/JNK, p-p38/p38, and IκB-α/GAPDH ratios. Total ERK1/2, JNK, and p38 served as corresponding controls, and GAPDH served as a loading control. Results are presented as the mean ± standard deviation (SD) of three independent experiments. *, compared with the 0 min group; †, compared with the Saline + LPS group. */† *p* < 0.05.

The effects of the Hongdan extract on MAPK and NF-κB signaling pathways during LPS-induced inflammatory responses in BV2 microglia cells were examined. In the LPS-treated group, the ratios of p-ERK/ERK, p-JNK/JNK, and p-p38/p38 increased in a time-dependent manner, indicating activation of inflammatory signaling. In contrast, these ratios were significantly reduced in the Hongdan + LPS group compared with the LPS-only group, demonstrating that the Hongdan extract effectively suppressed activation of inflammatory signaling pathways. However, the expression of IκB-α, a key regulator of the NF-κB pathway, was not altered by the Hongdan extract, suggesting that the inhibitory effect was more closely associated with MAPK signaling than with detectable changes in IκB-α under the present experimental conditions (Figure 10).

## 3. Discussion

Neurodegenerative diseases, including Alzheimer’s disease, Parkinson’s disease, Huntington’s disease, and related progressive disorders, are characterized by a gradual loss of neuronal structure and function that ultimately leads to synaptic failure, neuronal death, and progressive cognitive or motor impairment [29]. Increasing evidence indicates that chronic neuroinflammation is not merely a secondary consequence but a central driving force in disease progression, mediated largely by persistent activation of microglia and sustained production of inflammatory mediators [30].

*Salvia miltiorrhiza* is a widely used medicinal herb whose roots contain lipophilic tanshinones and hydrophilic phenolic acids, including salvianolic acid B, which have been associated with antioxidant, anti-inflammatory, and neuroprotective activities [16,17]. For example, salvianolic acid B suppresses microglial activation and inflammatory responses, contributing to neuroprotection [31], while whole extracts of *Salvia miltiorrhiza* have shown efficacy in cellular models of amyloid-β-induced neuronal injury and in animal models of cerebral ischemia and neurodegeneration [20]. Additionally, pharmacological analyses suggest that *Salvia miltiorrhiza*-derived compounds may serve as candidates for anti-Alzheimer’s therapeutics through multi-target mechanisms [32]. Recent systems pharmacology studies further support this multi-target paradigm. Network pharmacology analyses of *Salvia miltiorrhiza* have identified multiple disease-relevant signaling networks associated with neuroinflammation, neuronal survival, and neuroprotection as major nodes of therapeutic action [33,34,35]. These pathways are critically implicated in microglia-mediated neuroinflammation and neuronal damage, reinforcing the biological plausibility of *Salvia miltiorrhiza* as a disease-modifying agent.

Although substantial evidence has accumulated regarding the neuroprotective and anti-inflammatory effects of isolated constituents from *Salvia miltiorrhiza*, comparatively fewer studies have evaluated standardized extracts derived from newly developed cultivars, particularly in microglial models. In this context, ‘Hongdan’ represents a significant advancement in the cultivation of *Salvia miltiorrhiza*, characterized by its superior phytochemical profile compared to conventional reference cultivars. Previous comparative analyses have demonstrated that ‘Hongdan’ contains approximately 3-fold higher levels of salvianolic acid B and tanshinone IIA than the widely established reference cultivar ‘ZD1’, along with significantly elevated total phenolic and terpenoid contents [26,27,28]. This high-content characteristic suggests that ‘Hongdan’ could serve as a more potent and standardized source for therapeutic applications. BV2 microglial cells are commonly used as an in vitro model of activated microglia because LPS stimulation induces robust production of nitrite and pro-inflammatory cytokines through MAPK and NF-κB signaling pathways, closely mimicking inflammatory processes observed in neurodegenerative disorders [6]. Therefore, the present study aimed to evaluate the anti-neuroinflammatory effects of a 50% ethanol extract prepared from this high-potency Korean cultivar, *Salvia miltiorrhiza* ‘Hongdan’, in LPS-stimulated BV2 microglial cells and to elucidate its potential mechanism of action involving the MAPK and NF-κB signaling pathways.

Excessive and uncontrolled activation of microglia results in the overproduction of inflammatory mediators, including iNOS and COX-2, as well as pro-inflammatory cytokines such as IL-1β, IL-6, and TNF-α. This sustained neuroinflammatory milieu contributes to neuronal dysfunction and the progression of neurodegenerative disorders [6]. In particular, excessive nitrite production during inflammatory conditions is primarily mediated by iNOS, distinguishing it from constitutive NO generation involved in normal physiological regulation. Similarly to iNOS, COX-2 is an inducible enzyme whose expression is markedly increased by inflammatory stimuli such as LPS, cytokines, and chemokines. Overproduction of COX-2 leads to elevated synthesis of pro-inflammatory prostaglandins, thereby contributing to the progression of inflammatory processes [36]. In this study, the Hongdan extract attenuated LPS-induced nitrite production and the upregulated expression of iNOS and COX-2 in BV2 microglia, indicating effective suppression of inflammatory mediator production. In addition, LPS-induced mRNA expression of pro-inflammatory cytokines, including IL-1β and IL-6, was markedly reduced in a dose-dependent manner, whereas TNF-α expression was significantly suppressed only at the highest concentration tested. These findings suggest that the Hongdan extract attenuates microglia-mediated pro-inflammatory cytokine production. The MAPK signaling pathway plays a critical role in regulating cellular processes such as proliferation, differentiation, apoptosis, and immune responses [37]. It comprises three major kinase cascades: ERK, JNK, and p38, whose activation has been closely associated with inflammatory responses [38]. NF-κB is another key transcription factor that regulates the expression of inflammatory mediators and cytokines through degradation of its inhibitory protein IκB-α [39]. In the present study, LPS stimulation markedly increased the phosphorylation of ERK, JNK, and p38 in BV2 microglial cells, indicating activation of inflammatory signaling pathways. Treatment with the Hongdan extract significantly suppressed the phosphorylation of these MAPK components, whereas the degradation of IκB-α was not affected.

These findings are largely consistent with previous studies reporting anti-inflammatory effects of bioactive constituents from *Salvia miltiorrhiza*. Tanshinone IIA has been shown to suppress iNOS expression and the production of pro-inflammatory cytokines such as TNF-α, IL-1β, and IL-6 in activated macrophages [40]. Similarly, salvianolic acid B was reported to inhibit LPS-induced expression of iNOS, TNF-α, and IL-1β at both transcriptional and protein levels, thereby attenuating microglial activation [31]. The Hongdan extract suppressed LPS-induced nitrite production, iNOS and COX-2 expression, and pro-inflammatory cytokine expression in BV2 microglia, indicating a comparable anti-neuroinflammatory profile. However, unlike the dose-dependent inhibition of TNF-α observed in some previous studies, TNF-α expression in the present study was reduced only at the highest concentration. This discrepancy may be attributable to differences in experimental systems, including the use of BV2 immortalized microglial cells rather than primary microglia, as well as variations in extract composition, concentration range, and signaling pathways targeted by the complex mixture of constituents present in the Hongdan extract. The inhibitory effects of the Hongdan extract on MAPK activation observed in this study are consistent with previous reports demonstrating that *Salvia miltiorrhiza* preparations modulate ERK, JNK, and p38 MAPK signaling pathways in various experimental models [41,42,43]. Herbal extracts containing *Salvia miltiorrhiza* have been shown to regulate MAPK-dependent signaling in both in vitro and in vivo models, supporting the role of MAPK modulation as an important mechanism underlying their biological activities [44]. However, the influence of *Salvia miltiorrhiza*-derived compounds on NF-κB signaling appears to be more variable. For example, salvianolic acid B has been reported to suppress NF-κB activation and reduce inflammatory mediator production in primary microglia [31]. In contrast, other studies using complex extracts of *Salvia miltiorrhiza* have demonstrated inhibition of MAPK signaling without significant effects on NF-κB activation or IκB-α degradation [45]. In the present study, the Hongdan extract markedly suppressed LPS-induced phosphorylation of ERK, JNK, and p38, whereas IκB-α degradation was not altered by the extract, suggesting that its anti-inflammatory effects are more closely associated with inhibition of MAPK signaling than with detectable suppression of the canonical IκB-α-dependent NF-κB pathway. Collectively, our results support the anti-inflammatory activity of the Hongdan extract in LPS-stimulated BV2 microglia under the present experimental conditions. However, to further support its development as a natural therapeutic candidate for neuroinflammatory disorders, future studies should evaluate whether its active constituents or metabolites can overcome blood–brain barrier-related pharmacokinetic limitations through approaches such as lipophilic or tanshinone-enriched fractionation, bioavailability-enhancing formulations, and LC-MS/MS-based pharmacokinetic and brain-distribution analyses.

## 4. Materials and Methods

### 4.1. Sample Preparation

Root samples of the *Salvia miltiorrhiza* cultivar ‘Hongdan’ were obtained from the National Institute of Horticultural and Herbal Science, RDA, Republic of Korea. The source plant materials originated from cultivation lots previously described by Han et al. [28]. For the present study, lateral roots were removed, and the main roots were cut into segments of approximately 5 cm, followed by hot-air drying at 55 °C for 12 h. The dried root material (2.0 kg) was pulverized and extracted twice under reflux with 10 volumes of 50% ethanol at 80 °C for 4 h per cycle. The combined extracts were concentrated under reduced pressure at 35 °C, stored at −80 °C for 12–24 h and freeze-dried. The dried extract was finely powdered and stored at 4 °C until use.

### 4.2. Reagents and Instruments

Fetal bovine serum (FBS), RPMI 1640 medium, and antibiotics were purchased from Gibco BRL (Grand Island, NE, USA). 1-Methyl-4-phenyl-1,2,3,6-tetrahydropyridine (MPTP), chloroform, premade acrylamide gel solution, sodium dodecyl sulfate (SDS), lipopolysaccharide (LPS), Tris-HCl, 3-(4,5-dimethylthiazol-2-yl)-2,5-diphenyl-2H-tetrazolium bromide (MTT), dimethyl sulfoxide (DMSO), Griess reagent, phosphate-buffered saline (PBS) solution, and 2-propanol were obtained from Sigma (St. Louis, MO, USA). All antibodies used for Western blotting were purchased from Cell Signaling Technology (Danvers, MA, USA).

### 4.3. Determination of Total Phenolic Content (TPC)

Total phenolic content was determined using a modified Folin–Ciocalteu method [46]. The Hongdan extract was diluted to 1 mg/mL, and 20 μL of the sample was mixed with 100 μL of Folin–Ciocalteu phenol reagent and 80 μL of 7.5% Na_2_CO_3_ solution. The mixture was incubated in the dark at room temperature for 45 min, and absorbance was measured at 760 nm using a microplate reader. A standard calibration curve was generated using gallic acid, and the results were expressed as μg gallic acid equivalents (GAE)/mL.

### 4.4. Determination of Total Flavonoid Content (TFC)

Total flavonoid content was determined using a previously reported method [47], with slight modifications. The Hongdan extract was diluted to 5 mg/mL, and 20 μL of the sample was mixed with 60 μL of 95% ethanol, 4 μL of 10% Al_2_(CO_3_)_3_·6H_2_O, 4 μL of 1.0 M CH_3_COOK, and 112 μL of distilled water. The mixture was incubated at room temperature for 40 min, after which the absorbance was measured at 415 nm using a microplate reader. A standard calibration curve was prepared using quercetin, and the results were expressed as μg quercetin equivalents (QE)/mL.

### 4.5. Cell Culture

BV2 microglial cells were used for the cell experiments and were kindly provided by the College of Korean Medicine, Wonkwang University. The BV2 cell line was cultured in RPMI 1640 medium supplemented with 10% fetal bovine serum (FBS) and 1% penicillin–streptomycin. Cells were maintained at 37 °C in a humidified atmosphere containing 5% CO_2_. Prior to experiments, cultures were maintained at 70–80% confluence.

### 4.6. MTT Assay

Cell viability was assessed using the MTT assay [48]. BV2 microglial cells were seeded at a density of 2.0 × 10^5^ cells/mL and treated with various concentrations of the Hongdan extract (50, 100, 200, 300, and 500 ng/mL) for 24 h. MTT solution was added to each well and incubated for 30 min. The resulting formazan crystals were dissolved in DMSO, and absorbance was measured at 450 nm using a spectrophotometer (Molecular Devices, San Jose, CA, USA).

### 4.7. Measurement of Nitrite Production

BV2 microglial cells were pretreated with the Hongdan extract (50, 100, 200, and 300 ng/mL) for 1 h and then stimulated with LPS (1 μg/mL) for 24 h. To determine nitrite production, the culture supernatants were mixed with Griess reagent (1% sulfanilamide, 0.1% N-(1-naphthyl) ethylenediamine dihydrochloride, and 45% phosphoric acid) and incubated at room temperature [49]. Absorbance was measured at 540 nm using a spectrophotometer (Molecular Devices, San Jose, CA, USA). Nitrite production was calculated from the absorbance values.

### 4.8. RNA Extraction and Quantitative PCR

To evaluate the expression of inflammation-related genes, total RNA was isolated and quantitative real-time reverse transcription polymerase chain reaction (qRT-PCR) was performed [50]. BV2 cells were seeded in 6-well plates at a density of 1 × 10^6^ cells/well. Cells were pretreated with the Hongdan extract (50, 100, 200, and 300 ng/mL) for 1 h and then stimulated with LPS (1 μg/mL) for 6 h. After removing the supernatant, RNA was extracted using Easy-Blue RNA (iNtRON Biotechnology, Seongnam, Republic of Korea), and purity was confirmed using a GeneQuant Pro RNA Calculator (Biochrom, Cambridge, UK). Cells were washed twice with PBS, collected in 1 mL PBS, and centrifuged. The pellet was lysed by adding 1 mL Easy-Blue solution. Chloroform (100 μL) was added, and the samples were centrifuged at 15,000 rpm for 15 min to separate the aqueous phase. An equal volume of 2-propanol was added, followed by centrifugation at 15,000 rpm for 10 min. The RNA pellet was washed twice with 80% ethanol, air-dried, and dissolved in 15 μL of diethyl pyrocarbonate (DEPC)-treated water. cDNA was synthesized from 1 μg of total RNA using a cDNA synthesis kit (Applied Biosystems, Carlsbad, CA, USA). Quantitative PCR was performed using 1 μL of cDNA, 4 μL of real-time PCR master mix, and gene-specific primers. PCR cycling conditions were as follows: 92 °C for 30 s, 60 °C for 40 s, and 72 °C for 30 s, repeated for 40 cycles. Forward and reverse primers were synthesized by Cosmo Genetech (Daejeon, Republic of Korea). The primer sequences are listed in Table 2.

### 4.9. Western Blot Analysis

BV2 microglial cells were seeded at a density of 5.0 × 10^6^ cells per 6 cm dish and pretreated with the Hongdan extract (300 ng/mL) for 1 h, followed by stimulation with lipopolysaccharide (LPS, 1 μg/mL) for 0, 15, 30, or 60 min. After treatment, the cells were washed three times with cold PBS and centrifuged at 5000 rpm for 5 min to remove the supernatant. The resulting cell pellets were collected and lysed with RIPA lysis buffer (1 mL RIPA buffer supplemented with 10 μL phosphatase inhibitor and 10 μL protease inhibitor). The lysates were then centrifuged at 15,000 rpm for 20 min to remove insoluble debris, and the protein concentration was determined. Equal amounts of protein were mixed with 4× sample buffer, separated by 10% SDS–PAGE, and transferred onto membranes. The membranes were blocked with 5% skim milk for 2 h and subsequently used for immunoblotting. Protein expression was detected using an enhanced chemiluminescence (ECL) detection system (Amersham, UK) [51].

### 4.10. Statistical Analysis

All experiments were performed at least three times independently, and the results are expressed as the mean ± standard deviation (SD). Statistical significance was analyzed using SPSS for Windows (version 26.0; IBM Corp., Armonk, NY, USA). Data were evaluated by one-way analysis of variance (ANOVA), followed by Tukey’s post hoc test. Differences were considered statistically significant at *p* < 0.05.

## 5. Conclusions

This study demonstrated that a 50% ethanol extract of *Salvia miltiorrhiza* ‘Hongdan’ effectively attenuates LPS-induced inflammatory responses in BV2 microglial cells. The Hongdan extract significantly suppressed nitrite production, reduced the expression of iNOS and COX-2, and downregulated pro-inflammatory cytokines, including IL-1β and IL-6, with partial inhibition of TNF-α at higher concentrations. These anti-inflammatory effects were associated with inhibition of MAPK signaling pathways, while canonical NF-κB regulation appeared less affected under the present conditions. Collectively, these findings suggest that Hongdan may represent a promising medicinal resource with potential for development as a reproducible anti-neuroinflammatory agent. Further studies using in vivo models will be required to clarify the underlying mechanisms and therapeutic relevance.

## Figures and Tables

**Table 1:** Salvianolic acid B and tanshinone IIA contents and its yield of *Salvia miltiorrhiza* ‘Hongdan’. kg/10a represents kilograms per 10 ares (1000 m^2^).

Variety	Salvianolic Acid B		Tanshinone IIA		Total
	mg/g	kg/10a	mg/g	kg/10a	mg/g
Hongdan	72.35 ± 1.39	18.70 ± 0.36	3.80 ± 0.07	0.98 ± 0.02	76.15 ± 1.47

Adapted from Han et al., 2024 [28].

**Table 2:** The Primer of iNOS, COX-2, IL-1β, IL-6, TNF-α, and GAPDH.

Gene	Primer
iNOS	F: 5′-GTTGAAGACTGAGACTCTGG-3′
	R: 5′-GACTAGGCTACTCCGTGGA-3′
COX-2	F: 5′-GGTGGCTGTTTTGGTAGG CTG-3′
	R: 5′-GGGTTGCTGGGGGAAGAAATG-3′
IL-1β	F: 5′-CCTCGTGCTGTCGGACCCAT-3′
	R: 5′-CAGGCTTGTGCTCTGCTTGTGA-3′
IL-6	F: 5′-CCGGAGAGGAGACTTCACAG-3′
	R: 5′-CAGAATTGCCATTGCACAAC-3′
TNF-α	F: 5′-GTGGAACTGGCAGAAGAGGC-3′
	R: 5′-AGACAGAAGAGCGTGGTGGC-3′
GAPDH	F: 5′-TGTGTCCGTCGTGGATCTGA-3′
	R: 5′-TTGCTGTTGAAGTCGCAGGAG-3′

## Data Availability

The original contributions presented in this study are included in the article. Further inquiries can be directed to the corresponding author.

## Abbreviations

The following abbreviations are used in this manuscript:

COX-2	Cyclooxygenase-2
DMSO	Dimethyl sulfoxide
ERK1/2	Extracellular signal-regulated kinase 1/2
FBS	Fetal bovine serum
GAE	Gallic acid equivalents
GAPDH	Glyceraldehyde-3-phosphate dehydrogenase
HPLC	High-performance liquid chromatography
IκB-α	Inhibitor of kappa B alpha
IL-1β	Interleukin-1 beta
IL-6	Interleukin-6
iNOS	Inducible nitric oxide synthase
JNK	c-Jun N-terminal kinase
LPS	Lipopolysaccharide
MAPK	Mitogen-activated protein kinase
MTT	3-(4,5-dimethylthiazol-2-yl)-2,5-diphenyl-2H-tetrazolium bromide
NF-κB	Nuclear factor kappa B
PBS	Phosphate-buffered saline
QC	Quality control
qRT-PCR	Quantitative real-time reverse transcription polymerase chain reaction
QE	Quercetin equivalents
RDA	Rural Development Administration
RIPA	Radioimmunoprecipitation assay
RNA	Ribonucleic acid
RPMI	Roswell Park Memorial Institute medium
SDS	Sodium dodecyl sulfate
TFC	Total flavonoid content
TNF-α	Tumor necrosis factor alpha
TPC	Total phenolic content
